# Omega-6 sparing effects of parenteral lipid emulsions—an updated systematic review and meta-analysis on clinical outcomes in critically ill patients

**DOI:** 10.1186/s13054-022-03896-3

**Published:** 2022-01-19

**Authors:** Quirin Notz, Zheng-Yii Lee, Johannes Menger, Gunnar Elke, Aileen Hill, Peter Kranke, Daniel Roeder, Christopher Lotz, Patrick Meybohm, Daren K. Heyland, Christian Stoppe

**Affiliations:** 1 Department of Anesthesiology, Intensive Care, Emergency and Pain Medicine, University Hospital Wuerzburg, Wuerzburg, Germany; 2 Department of Anesthesiology, University of Malaya, Kuala Lumpur, Malaysia; 3 Department of Anesthesiology and Intensive Care Medicine, University Medical Center Schleswig-Holstein, Campus Kiel, Kiel, Germany; 4 Department of Anesthesiology and Intensive Care Medicine, University Hospital RWTH Aachen, Aachen, Germany; 5 Department of Critical Care Medicine, Queen’s University, Kingston, Canada; 6 Clinical Evaluation Research Unit, Kingston General Hospital, Kingston, Canada

**Keywords:** Omega-6 fatty acid, Fish oil, Omega-3 fatty acid, Immunonutrition, Critical illness, Parenteral nutrition

## Abstract

**Background:**

Parenteral lipid emulsions in critical care are traditionally based on soybean oil (SO) and rich in pro-inflammatory omega-6 fatty acids (FAs). Parenteral nutrition (PN) strategies with the aim of reducing omega-6 FAs may potentially decrease the morbidity and mortality in critically ill patients.

**Methods:**

A systematic search of MEDLINE, EMBASE, CINAHL and CENTRAL was conducted to identify all randomized controlled trials in critically ill patients published from inception to June 2021, which investigated clinical omega-6 sparing effects. Two independent reviewers extracted bias risk, treatment details, patient characteristics and clinical outcomes. Random effect meta-analysis was performed.

**Results:**

1054 studies were identified in our electronic search, 136 trials were assessed for eligibility and 26 trials with 1733 critically ill patients were included. The median methodologic score was 9 out of 14 points (95% confidence interval [CI] 7, 10). Omega-6 FA sparing PN in comparison with traditional lipid emulsions did not decrease overall mortality (20 studies; risk ratio [RR] 0.91; 95% CI 0.76, 1.10; *p* = 0.34) but hospital length of stay was substantially reduced (6 studies; weighted mean difference [WMD] − 6.88; 95% CI − 11.27, − 2.49; *p* = 0.002). Among the different lipid emulsions, fish oil (FO) containing PN reduced the length of intensive care (8 studies; WMD − 3.53; 95% CI − 6.16, − 0.90; *p* = 0.009) and rate of infectious complications (4 studies; RR 0.65; 95% CI 0.44, 0.95; *p* = 0.03). When FO was administered as a stand-alone medication outside PN, potential mortality benefits were observed compared to standard care.

**Conclusion:**

Overall, these findings highlight distinctive omega-6 sparing effects attributed to PN. Among the different lipid emulsions, FO in combination with PN or as a stand-alone treatment may have the greatest clinical impact.

*Trial registration* PROSPERO international prospective database of systematic reviews (CRD42021259238).

**Graphical abstract:**

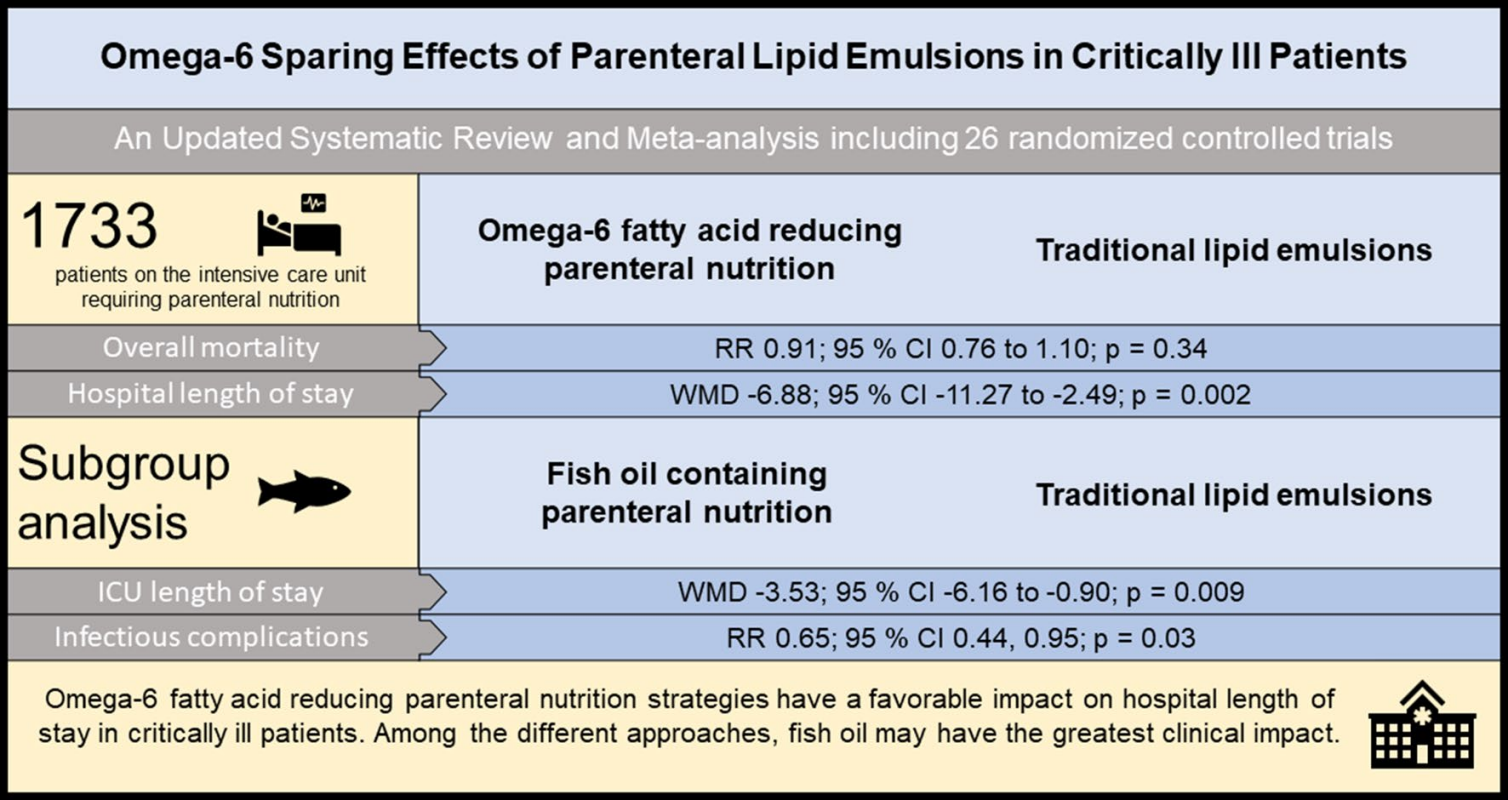

**Supplementary Information:**

The online version contains supplementary material available at 10.1186/s13054-022-03896-3.

## Background

Critical illness is often characterized by an imbalanced immune response, which can lead to excessive cytokine release and accumulation of reactive oxygen species. Systemic inflammation and oxidative stress ultimately result in tissue damage, multi organ failure and high mortality rates [1–4]. Parenteral lipid emulsions provide fatty acids (FAs) as a source of calories and cellular building blocks [5] and have been under investigation because of their immunomodulating features [6]. Traditionally, parenteral lipid emulsions are derived from plant—and especially soybean oil (SO) to provide the patient with essential long-chain triglycerides (LCTs). However, SO’s ratio of omega-6 polyunsaturated FAs to omega-3 polyunsaturated FAs (7:1) is being regarded critically [6]. In vivo, linoleic acid (omega-6) is converted to arachidonic acid and pro-inflammatory eicosanoids, such as prostaglandins, thromboxanes and leukotrienes. On the other hand, α-linolenic acid (omega-3) is the metabolic precursor of docosahexaenoic acid (DHA) and eicosapentaenoic acid (EPA), which both have anti-inflammatory and anti-oxidative properties like inhibitory effects on various innate and adaptive immune cells and the transcription of inflammatory cytokines [7–9]. Unfortunately, the pathway of α-linolenic acid conversion to DHA and EPA appears to be highly ineffective in most humans [10] and an abundance of omega-6 FAs will furthermore suppress the balancing effects of omega-3 FA conversion, as both FAs (omega-3 and omega-6) compete for the same enzymes [11]. Therefore, omega-6 FA reducing strategies have been introduced to clinical nutrition. A reduction in omega-6 FAs can be achieved with the addition of medium-chain triglycerides (MCT), olive oil (OO) or fish oil (FO) to SO-based lipid emulsions. FO supplements, as a major source of DHA and EPA, can simultaneously provide adequate levels of omega-3 FAs, which is regarded as an attractive immunomodulatory treatment option with potential clinical benefits [12]. Hitherto, meta-analyses solely focused on the FO aspect, resulting in limited evidence regarding the overall picture of omega-6 FA reduction. After aggregating 10 randomized controlled trials (RCTs) in 2015, Manzanares et al. found that lipid emulsions with a FO component may reduce infectious complications, duration of mechanical ventilation (MV) and hospital length of stay (LOS) in critically ill patients [13]. In the most recent meta-analysis by Pradelli et al., FO containing parenteral nutrition (PN) again reduced the rate of infections and hospital as well as ICU LOS [14]. Current nutrition guidelines state that DHA and EPA (FO dose of 0.1–0.2 g/kg/d) can be provided in patients receiving PN. Ambiguous results regarding other omega-6 sparing strategies are delineated, however without a clear recommendation [15, 16]. In recent years, several new RCTs on omega-6 sparing effects in general and on FO-containing lipid emulsions in particular have been published. This systematic review and meta-analysis aims to give a broad and comprehensive update on the emerging topic.

## Methods

### Information sources

We searched MEDLINE, EMBASE, Cumulative Index to Nursing and Allied Health Literature (CINAHL), the Cochrane Central Register of Controlled Trials and the Cochrane Database of Systematic Reviews (CENTRAL) for all relevant RCTs published between January 1980 and June 2021 with the following keywords: “fat emulsion”, “lipid emulsion”, “lipid injectable emulsion”, “lipids”, “triglycerides”, “medium chain triglycerides”, “long chain triglycerides”, “polyunsaturated fatty acids”, “omega-3 fatty acids”, “omega-6 fatty acids”, “fish oil”, “olive oil”, “soybean oil”, “linoleic acid”, “linolenic acid”, “eicosapentaenoic acid”, “EPA”, “docosahexaenoic acid” and “DHA”. Our data acquisition was not limited to articles written in English.

In case of missing data, we contacted the authors and requested additional information. The literature research, selection and methodologic assessment of trials was performed independently by two researchers. Consensus was required to include the respective study in the meta-analysis.

### Eligibility criteria

Inclusion criteria were defined as follows:

1. Study design: RCT with a parallel group.
2. Study population: adults, ≥ 18 years of age. Critical illness was defined as admission to the intensive care unit (ICU), or in unclear cases, a mortality rate of ≥ 5% in the control group. Studies that mainly or exclusively enrolled patients admitted for elective and cancer surgery were excluded, even if they were admitted to the ICU for postoperative surveillance.
3. Intervention and control: Studies were classified in two categories and analysed independently. The first group only comprised PN trials. Omega-6 FA reduced formulations were compared with standard care lipid emulsions containing higher amounts of omega-6 FAs.
  In the second group, patients received standard parenteral and / or enteral nutrition. Here, intravenous FO was administered as a stand-alone intervention. For control, patients either received no additional treatment or normal saline solution.
4. Outcomes: The primary outcome of our meta-analysis was overall mortality. When multiple mortality endpoints were reported in a trial, we included the data in the following order of preference: 28-day mortality > hospital mortality > ICU mortality > other mortality.

Secondary outcomes included 28-day mortality, ICU LOS, hospital LOS, days on MV and infectious complications. Trials without at least one of these clinical endpoints were excluded.

### Subgroup analysis

Among PN trials investigating omega-6 FA reduction, we predefined three subgroups according to the interventional strategy: SO/MCT, SO/OO and FO containing PN. To further reduce the heterogeneity of different FO formulations, we additionally aggregated all FO studies, which specifically tested Omegaven® (Fresenius Kabi, Bad Homburg, Germany), a 10% FO lipid emulsion with broad clinical use, and compared them to other FO solutions.

### Assessment of bias and methodologic quality

This systematic review adhered to the 2020 Preferred Reporting Items for Systematic Reviews and Meta-Analyses (PRISMA) statement [17]. As with our established practice in previous meta-analyses, studies were assigned to two categories based on the following criteria for methodologic quality: (1) concealed randomization, (2) blinded outcome adjudication and (3) intention-to-treat analysis. If all three characteristics applied, the study was considered “level I”, if one was unfulfilled we labeled the study as “level II”. In addition, the quality of each study was scored by two independent reviewers as described before [13, 18]. Nine items were considered: concealed randomization, intention-to-treat analysis, double blinding, consecutive patient selection, comparability at baseline, extent of follow-up, description of treatment protocols, description and equality of co-interventions and objective definition of outcomes. The maximum score was 14, the minimum score was 0 points (Additional file 1). The protocol of our meta-analysis was registered a priori at the PROSPERO international prospective database of systematic reviews (CRD42021259238).

### Data synthesis

All analyses were performed with a random effects model using RevMan 5.4 (Cochrane IMS, Oxford, UK). To estimate the pooled risk ratio (RR) for dichotomous data and the weighted mean difference (WMD) for continuous variables, data were aggregated from all studies and presented with a 95% confidence interval (CI). For continuous variables, we contacted the authors to provide the mean and standard deviation (SD), in case the original publication presented the median and interquartile range. If we were unable to obtain the mean and SD, the dataset was excluded from our analyses.

The *χ*^2^ test and the *I*^2^ statistics were used to assess heterogeneity. To address the risk of publication bias, a funnel plot was generated for the primary outcome and tested for asymmetry with the Egger regression test. As the overall purpose of this systematic review and meta-analysis was hypothesis generating, we considered a *p* value < 0.05 as statistically significant and a *p* value < 0.20 as a trend [19].

## Results

### Included trials

**Fig. 1:**
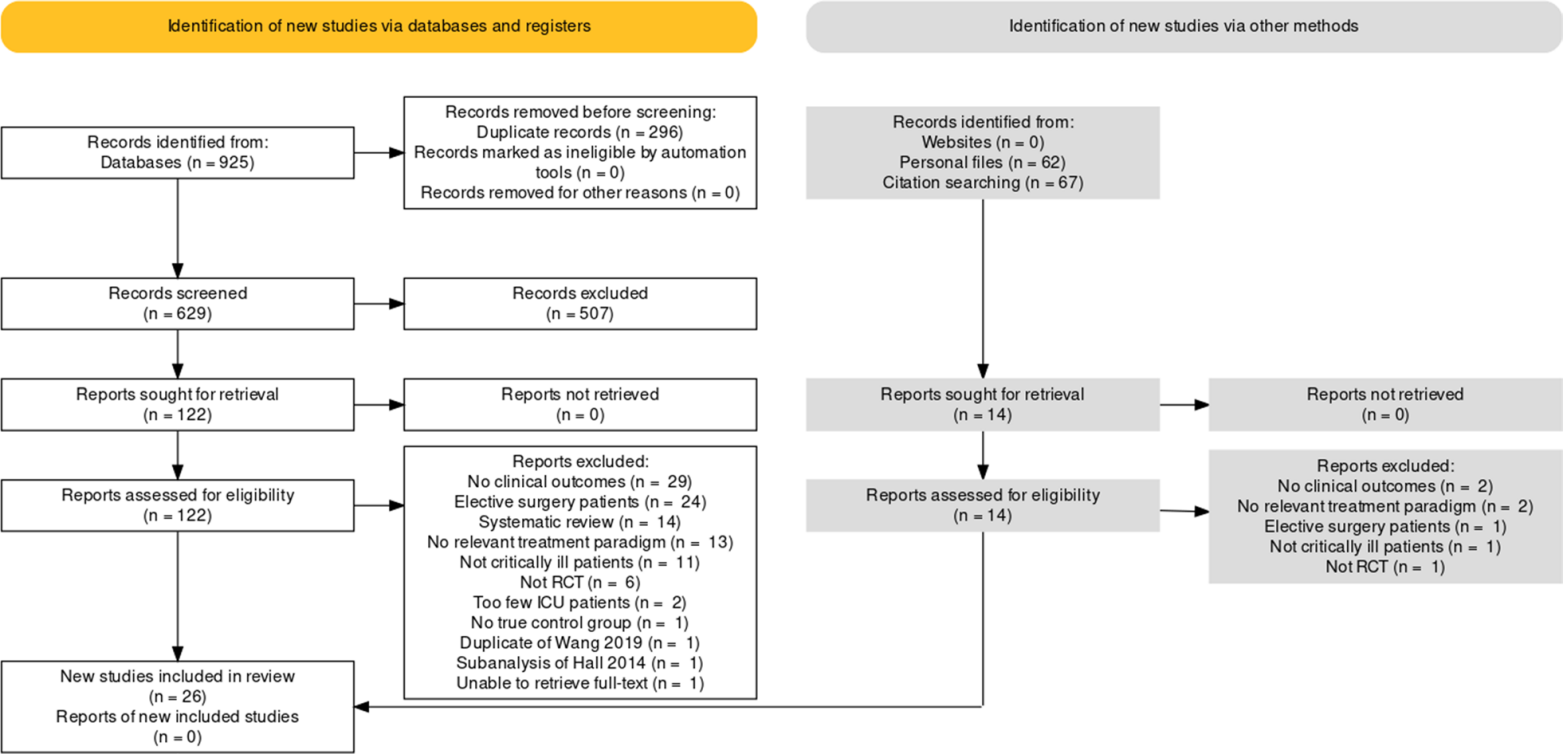
PRISMA flowchart delineating the process of trial inclusion

From the 1054 studies that were identified through our systematic searching, a total of 136 potential studies were sought for retrieval (Fig. 1). 40 trials covered non-ICU, elective surgery and cancer patients and 31 trials did not report on our clinical outcomes. 23 studies were not RCTs, including systematic reviews, meta- or sub-analyses. In 15 studies, the treatment paradigm did not fit the research question of our meta-analysis, mostly due to sole enteral nutrition. In one case a full text of the article could not be retrieved (Additional file 2). In the end, 26 trials with a total number of 1733 patients were included [20–45]. Five studies were labeled as “level I” and 21 studies as “level II”. The median methodologic score was 9 (95% CI 7, 10) for all included studies, 10 (95% CI 9, 14) for “Level I” trials and 9 (95% CI 6, 9) for “Level II” trials (Additional file 3). Eggers' test did not indicate the presence of a funnel plot asymmetry for our primary outcome (*p* = 0.77).

### Omega-6 fatty acid reducing strategies

**Fig. 2:**
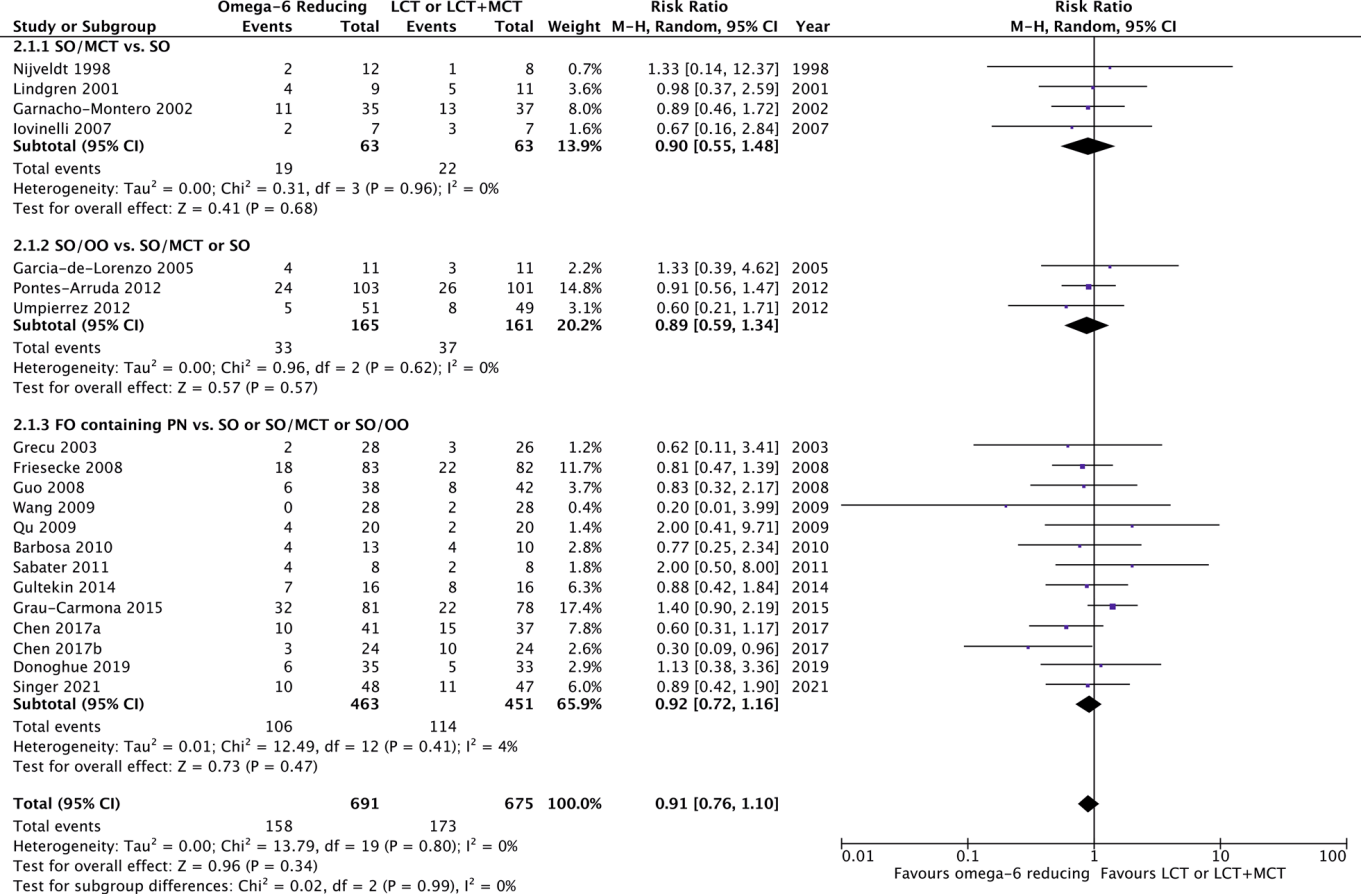
Overall mortality in trials using an omega-6 fatty acid reducing strategy

**Fig. 3:**
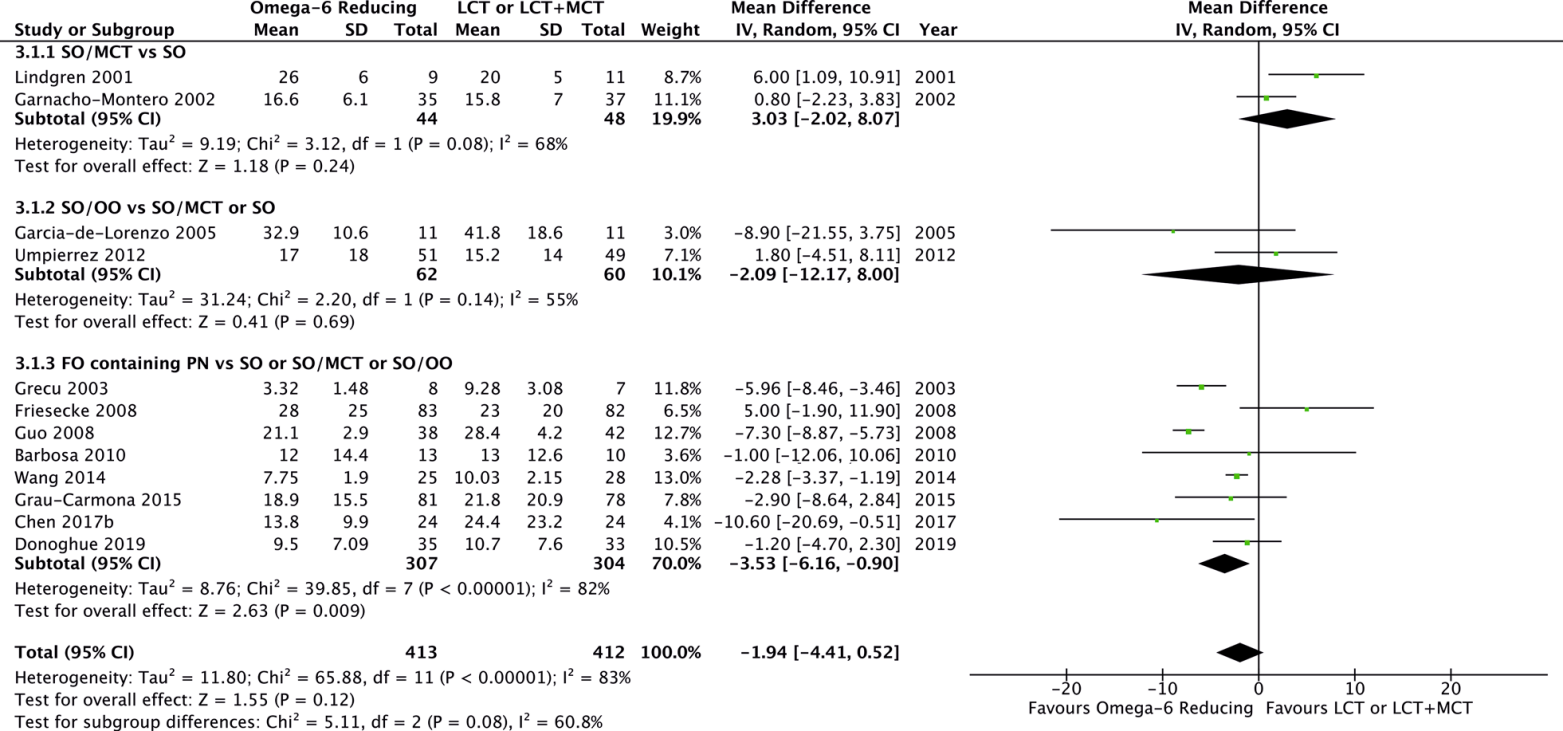
Intensive care unit length of stay in trials using an omega-6 fatty acid reducing strategy

There was no effect of omega-6 FA sparing PN in comparison to LCT or LCT/MCT on overall mortality (20 studies, *n* = 1366; RR 0.91; 95% CI 0.76, 1.10; *p* = 0.34; *I*^2^ = 0%; Fig. 2). However, we observed a trend towards lower 28-day mortality (8 studies, *n* = 733; RR 0.79; 95% CI 0.61, 1.02; *p* = 0.07; *I*^2^ = 0%; Additional file 4) and shorter ICU LOS (12 studies, *n* = 825; WMD − 1.94; 95% CI − 4.41, 0.52; *p* = 0.12; *I*^2^ = 83%; Fig. 3).

**Fig. 4:**
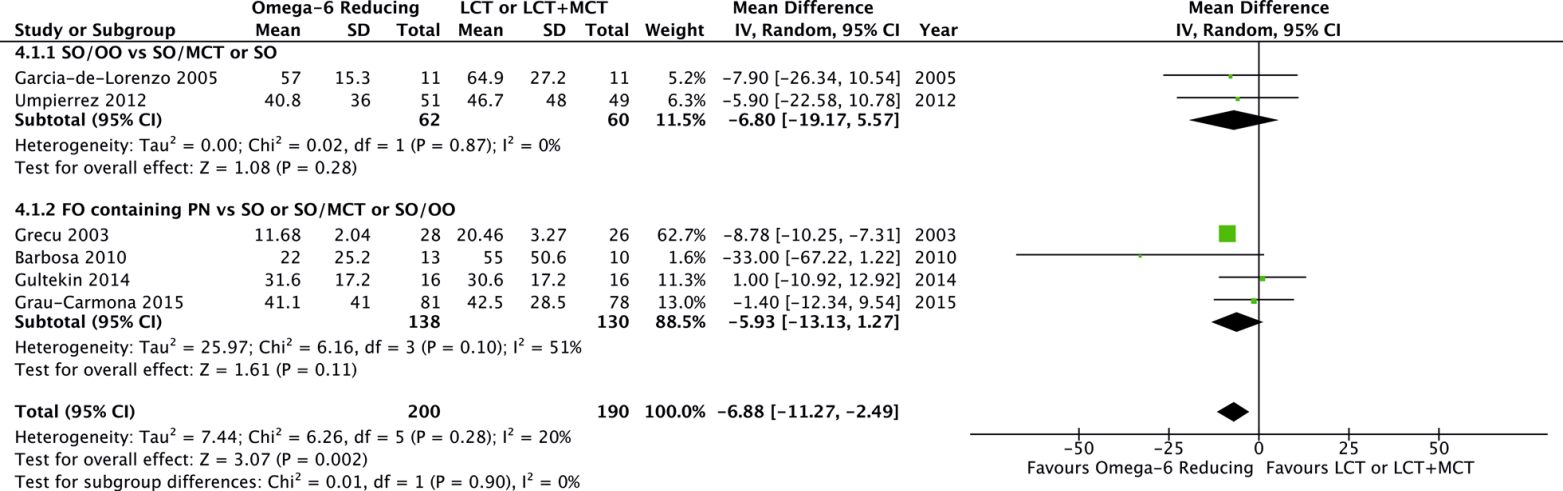
Hospital length of stay in trials using an omega-6 fatty acid reducing strategy

**Fig. 5:**
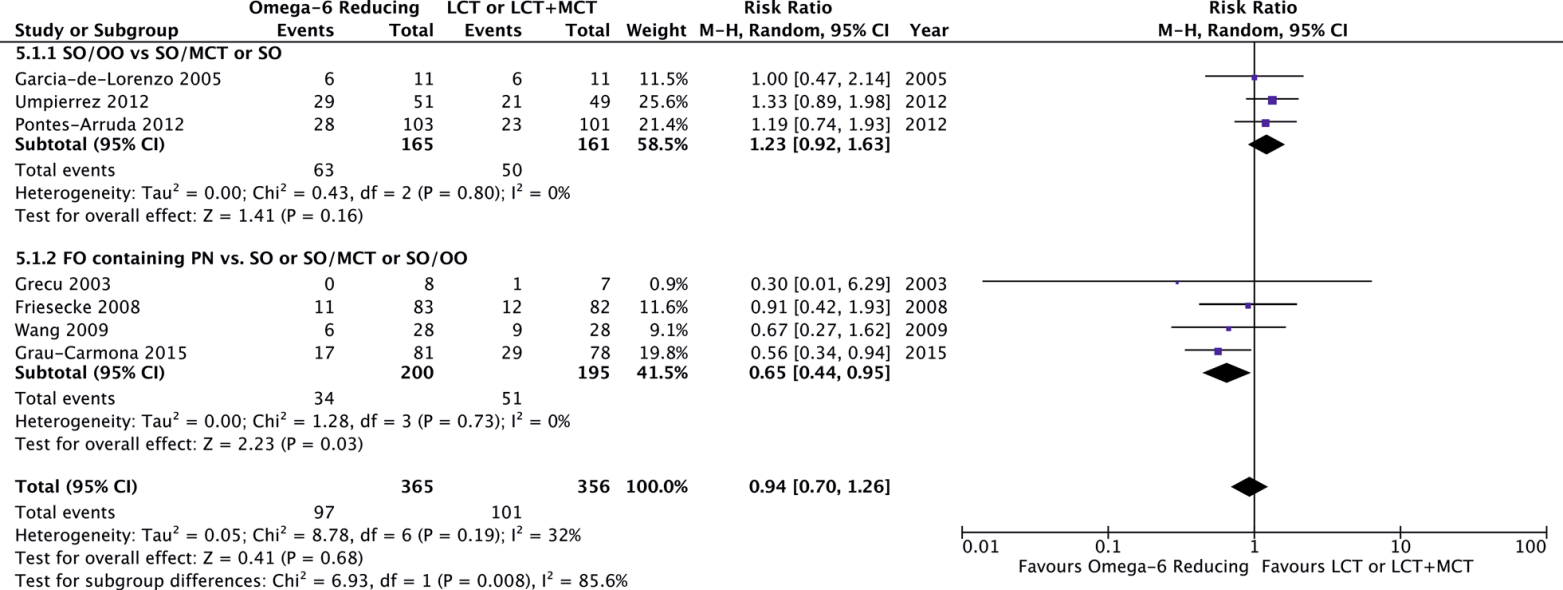
Infectious complications in trials using an omega-6 fatty acid reducing strategy

Compared to LCT or LCT/MCT, omega-6 FA sparing strategies significantly reduced the hospital LOS (6 studies, *n* = 390; WMD − 6.88; 95% CI − 11.27, − 2.49; *p* = 0.002; *I*^2^ = 20%; Fig. 4) and tended toward a shorter length of MV (9 studies, *n* = 511; WMD − 0.87; 95% CI − 1.82, 0.07; *p* = 0.07; *I*^2^ = 52%; Additional file 5). No benefits on the development of infectious complications were observed (7 studies, *n* = 721; RR 0.94; 95% CI 0.7, 1.26; *p* = 0.68; *I*^2^ = 32%; Fig. 5).

### Subgroup analysis of omega-6 fatty acid reducing strategies

Omega-6 FA reducing strategies were further analysed in three subgroups, SO/MCT, SO/OO and FO containing PN. None of the strategies affected overall mortality in comparison with LCT or LCT/MCT (test for subgroup differences, *p* = 0.99; Fig. 2). For FO containing PN, aggregated data suggested a trend toward reduced 28-day mortality (7 studies, *n* = 529; RR 0.74; 95% CI 0.54, 1.01; *p* = 0.06; *I*^2^ = 0%; Additional file 4). While SO/MCT and SO/OO did not affect the ICU LOS, FO containing PN significantly reduced the duration of intensive care (8 studies, *n* = 611; WMD − 3.53; 95% CI − 6.16, − 0.90; *p* = 0.009; *I*^2^ = 82%; test for subgroup differences, *p* = 0.08; Fig. 3). We also observed a trend towards shorter hospital LOS for FO containing PN (4 studies, *n* = 268; WMD − 5.93; 95% CI − 13.13, 1.27; *p* = 0.11; *I*^2^ = 51%; Fig. 4). In terms of MV, a reduction was reported for SO/MCT compared to SO (2 studies, *n* = 34; WMD − 3.30; 95% CI − 5.39, − 1.21; *p* = 0.002; *I*^2^ = 0%). Data on MV were furthermore reported in one trial with a SO/OO strategy and in six studies investigating FO containing PN, however, without clear benefits (test for subgroup differences, *p* = 0.03; Additional file 5). While SO/OO strategies were potentially associated with a trend toward more infectious complications (3 studies, *n* = 326; RR 1.23; 95% CI 0.92, 1.63; *p* = 0.16; *I*^2^ = 0%), FO containing PN significantly decreased nosocomial infections (4 studies, *n* = 395; RR 0.65; 95% CI 0.44, 0.95; *p* = 0.03; *I*^2^ = 0%; test for subgroup differences, *p* = 0.008; Fig. 5).

Among FO-containing PN, we further identified two strategies based on the usage of Omegaven and non-Omegaven lipid emulsions. We observed an overall mortality benefit for Omegaven (6 studies, *n* = 433; RR 0.68; 95% CI 0.48, 0.95; *p* = 0.03; *I*^2^ = 0%), which was not present in trials using other FO emulsions (test for subgroup differences, *p* = 0.02; Additional file 6). There were no clear signals regarding other clinical outcomes neither for Omegaven nor other FO lipid emulsions.

### Fish oil as a stand-alone intervention

**Fig. 6:**
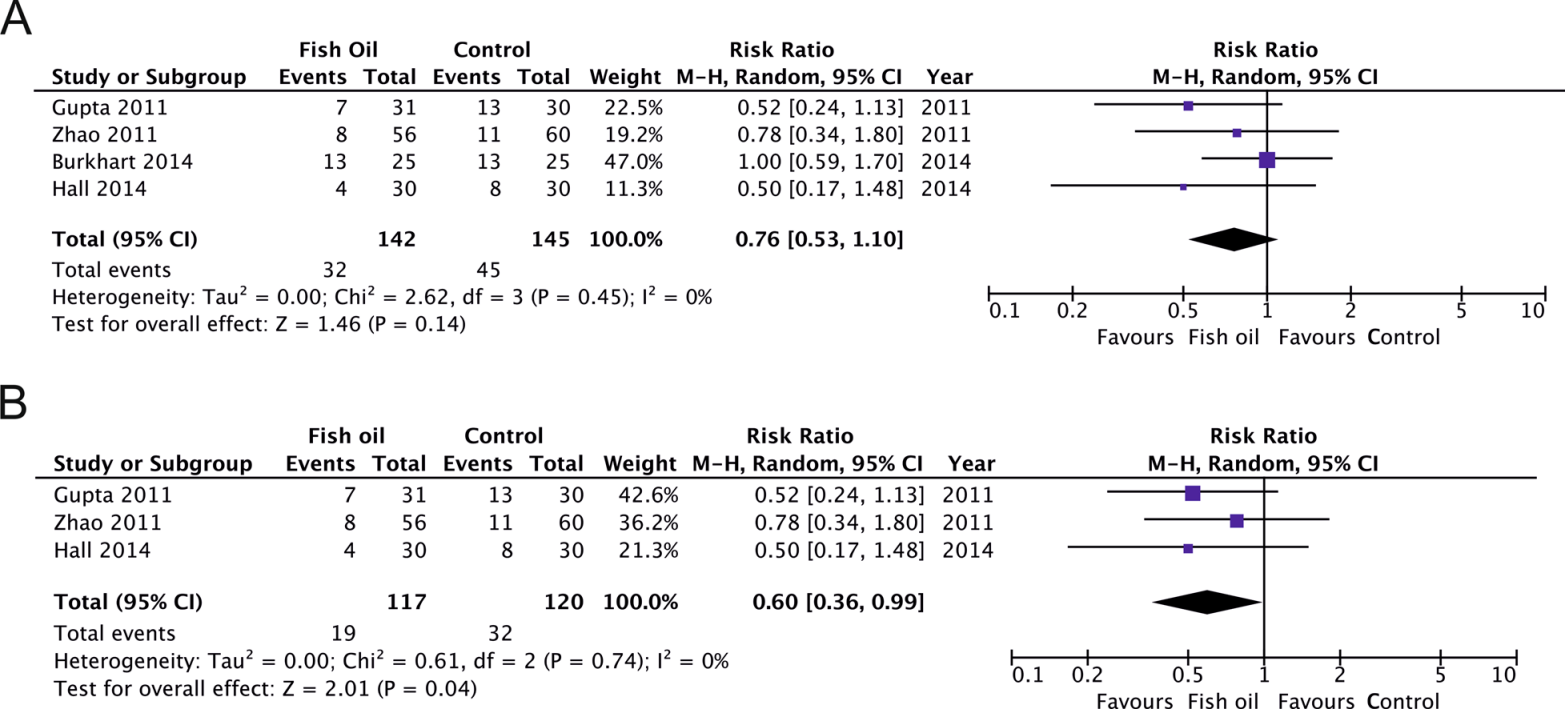
**A** Overall and **B** 28-day mortality in trials investigating stand-alone fish oil supplements

Five studies in patients on standard care nutrition compared a stand-alone FO intervention versus no additional supply of lipids. FO tended toward an improvement in overall mortality (4 studies, *n* = 287; RR 0.76; 95% CI 0.53, 1.10; *p* = 0.14; *I*^2^ = 0%) and significantly reduced 28-day mortality (3 studies, *n* = 237; RR 0.60; 95% CI 0.36, 0.99; *p* = 0.04; *I*^2^ = 0%; Fig. 6). Stand-alone FO was not associated with a shorter duration of intensive care (4 studies, *n* = 264; WMD − 1.38; 95% CI − 4.11, 1.34; *p* = 0.32; *I*^2^ = 52%) or hospital stay (3 studies, *n* = 148; WMD 0.78; 95% CI − 2.89, 4.46; *p* = 0.68; *I*^2^ = 0%). There was no aggregated data on MV and infectious complications available.

## Discussion

Overall, our systematic review and meta-analysis covered 26 trials in critically ill patients, of which 16 studies with 1046 patients were newly included in comparison with the previous meta-analysis by Manzanares et al. [13]. We hereby provide an update and additional insights not only on FO but also on general omega-6 FA reducing strategies in intensive care. Omega-6 sparing effects included a significant decrease in hospital LOS and trends towards reduction in 28-day mortality, ICU LOS and mechanical ventilation. Among different omega-6 FA reducing PN regimens, FO containing lipid emulsions reduced the length of intensive care and rate of nosocomial infections. Potential signals on mortality rates were observed with stand-alone use of FO, which encourages further research in this area.

In the past decades, clinical and technological advances promoting physiologic and metabolic resuscitation as well as early diagnosis and optimal treatment have significantly decreased hospital mortality in critical illness. In this context, nutritionally derived compounds exhibiting pharmacological effects contribute to an improved clinical outcome [46]. However, due to low quality of evidence, the potential benefits of anti-inflammatory and immunomodulating effects in critically ill patients remain unproven [47]. While a balance between pro- and anti-inflammatory response mechanisms is crucial for an adequate immune response, critically ill patients often exhibit immune dysregulation [48–50]. Imbalance arises from either excessive production of pro-inflammatory cytokines and reactive oxygen species or a lack of their physiological anti-inflammatory and anti-oxidative counterparts. A similar concept applies for omega-6 and omega-3 FAs or rather their metabolites. By reducing prostaglandins, leukotrienes and thromboxanes via MCT, OO or FO on the one hand and increasing DHA and EPA levels by FO on the other, pharmaconutrition might play a role in regaining inflammatory balance in critically ill patients. This basic concept is tempting, even though clinical evidence is scarce and ambiguous. There are—if at all—only minor effects of sole omega-6 FA reduction on inflammatory parameters and markers of oxidative stress [42, 51, 52], which is in line with our results, where the impact of SO/MCT and SO/OO on the clinical course was rather small. However, an RCT, in which OO was significantly associated with reduced ICU LOS and MV, had to be excluded, due to an unusual lipid- versus glucose-based treatment regimen [53]. In the case of FO, omega-6 sparing effects are complemented by a simultaneous increase in omega-3 FAs, which may be even more beneficial for inflammatory control.

In fact, FO has been studied for decades, after realizing that Inuits in Greenland with a fish-based diet had low rates of cardiovascular events [54]. This led to general recommendations for FO supplements in patients with coronary heart disease [55] and multiple promising investigations in autoimmune and inflammatory disorders [56–58]. Intravenous FO administration immediately affects physiological parameters, which was demonstrated by a significant increase in DHA and EPA concentrations in blood cell phospholipids within 60 min after infusion [59]. The rapid incorporation into various cellular membranes is crucial for an acute modulation of the immune response and suppression of pro-inflammatory cytokine release [60–62]. In addition, it has been recently discovered that DHA- and EPA-derived metabolites like resolvins, protectins, and maresins are required for the resolution of inflammation in the post-acute phase [7], as they regulate neutrophil recruitment and initiate macrophages to clear apoptotic cells and microorganisms [63].

Despite significant bias risk and rather small patient populations in the underlying studies [64–67], ESPEN guidelines state that parenteral FO can be provided for critically ill patients, as it likely decreases LOS and infections [16]. These conclusions align nicely with our results, including potential benefits on 28-day mortality and duration of MV. Hence, omega-6 FA reduction seems to hasten the recovery of patients requiring PN, which might justify the higher cost of these lipid emulsions in an economic evaluation. Pradelli et al. have recently conducted a systematic review and cost-effectiveness analysis on the use of omega-3 containing parenteral nutrition in ICU patients, which clearly demonstrated considerable cost savings due to a significant reduction in the risk of infections, and length of hospital or ICU stay. One main difference to our meta-analysis is that the authors included surgical patients without critical illness, for example postoperative surveillance after elective and cancer surgery [14]. Patterns of inflammation and the extent of organ injury might be very different in surgical patients with a defined tissue trauma, compared to systemic inflammation due to infection and sepsis. Our approach potentially provides a more homogenous collective of critically ill patients with a strong focus on sepsis and acute respiratory distress syndrome as well as additional insights into further omega-6 FA reducing strategies beyond FO. In comparison with Manzanares et al., we also excluded enteral nutrition protocols to further reduce heterogeneity [13]. Despite the distinctive differences between the meta-analyses, it is reassuring, that the results compare well.

We observe a trend towards improved 28-day survival, both for overall omega-6 FA reducing strategies as well as for FO containing PN alone. However, this potential signal could not be confirmed in terms of overall mortality. It has to be said, that multiple other factors and treatments beyond PN influence survival in critically ill patients, so that it is often difficult to interpret observed mortality benefits of a single intervention in modern ICU settings [68, 69]. In this light, the significant mortality benefits suggested for Omegaven and FO as a stand-alone medication have to be considered carefully.

We acknowledge several limitations of our meta-analysis. First, only five trials fulfilled the criteria of a “level I” study, which are concealed randomization, blinded outcome adjudication and intention-to-treat analysis. The median methodological score of all included studies was 9 out of 14 points, highlighting a considerable bias risk and mirroring the mediocre quality of most trials in the field of immunonutrition. This is a well-known problem in ICU research, as authors are faced with challenges of resources, recruitment and early randomization [70]. Second, the number of included studies and overall sample sizes for certain endpoints are too small to draw strong conclusions. Patient populations are still rather heterogeneous, despite our efforts to reduce variety. Third, overall mortality is an unsharp endpoint. Aggregating as many trials as possible comes at the price of including the whole variety of reported mortalities. Four studies for example only reported “other mortality” with a range from 15 to 109 days. Last, all included studies predominantly focused on “hard outcomes” and neglected the patient centered perspective. This requires more attention in future research.

On the other hand, this systematic review also has distinctive strengths. In contrast to previous meta-analyses, which merely focused on FO, we integrated additional omega-6 FA reducing PN strategies like SO/MCT and SO/OO [13, 14]. We only included RCTs with a relative specific patient population of critically ill ICU patients to reduce heterogeneity. We were not limited to any language barriers so that all internationally available evidence could be considered. Published abstracts, which did not undergo peer-review, were considered for inclusion after careful assessment. This approach might potentially reduce publication bias, we do, however, acknowledge the ongoing controversy on this topic [71–73]. We report the largest and the most current aggregation of PN lipid trials conducted in the critical care setting. To guarantee the reproducibility of this systematic review and meta-analysis, study eligibility decisions are provided in detail and data were abstracted using two independent experienced reviewers and referees.

## Conclusion

Taken together, the present meta-analysis in critically ill patients suggests omega-6 sparing effects attributed to parenteral immunonutrition. Among different lipid emulsions, clinical benefits were most pronounced for FO, either in combination with PN or as a stand-alone treatment.

## Supplementary Information

**Additional file 1**. Methodological quality scoring system

**Additional file 2**. List of excluded studies

**Additional file 3**. List of included studies, outcomes and bias risk assessment

**Additional file 4**. 28-day mortality in trials using an omega-6 fatty acid reducing strategy

**Additional file 5**. Length of mechanical ventilation in trials using an omega-6 fatty acid reducing strategy

**Additional file 6**. Overall mortality in trials using either Omegaven or other fish oil lipid emulsions

## Data Availability

Data generated or analysed during this study are included in this published article and its Additional files. Further information is available from the corresponding author on reasonable request.

## Abbreviations

CI: Confidence interval
DHA: Docosahexaenoic acid
EPA: Eicosapentaenoic acid
FA: Fatty acid
FO: Fish oil
ICU: Intensive care unit
LCT: Long-chain triglycerides
LOS: Length of stay
MCT: Medium-chain triglycerides
MV: Mechanical ventilation
OO: Olive oil
PN: Parenteral nutrition
RCT: Randomized controlled trial
RR: Risk ratio
SD: Standard deviation
SO: Soybean oil
WMD: Weighted mean difference
