# Supplementary material for: Omega-6 sparing effects of parenteral lipid emulsions—an updated systematic review and meta-analysis on clinical outcomes in critically ill patients
Source: Crit Care. 2022 Jan 19;26:23. doi: 10.1186/s13054-022-03896-3 (PMC8767697; doi:10.1186/s13054-022-03896-3)

**Supplement 1. Methodological Quality Scoring System (Canadian Nutrition Support Clinical Practice Guidelines)**


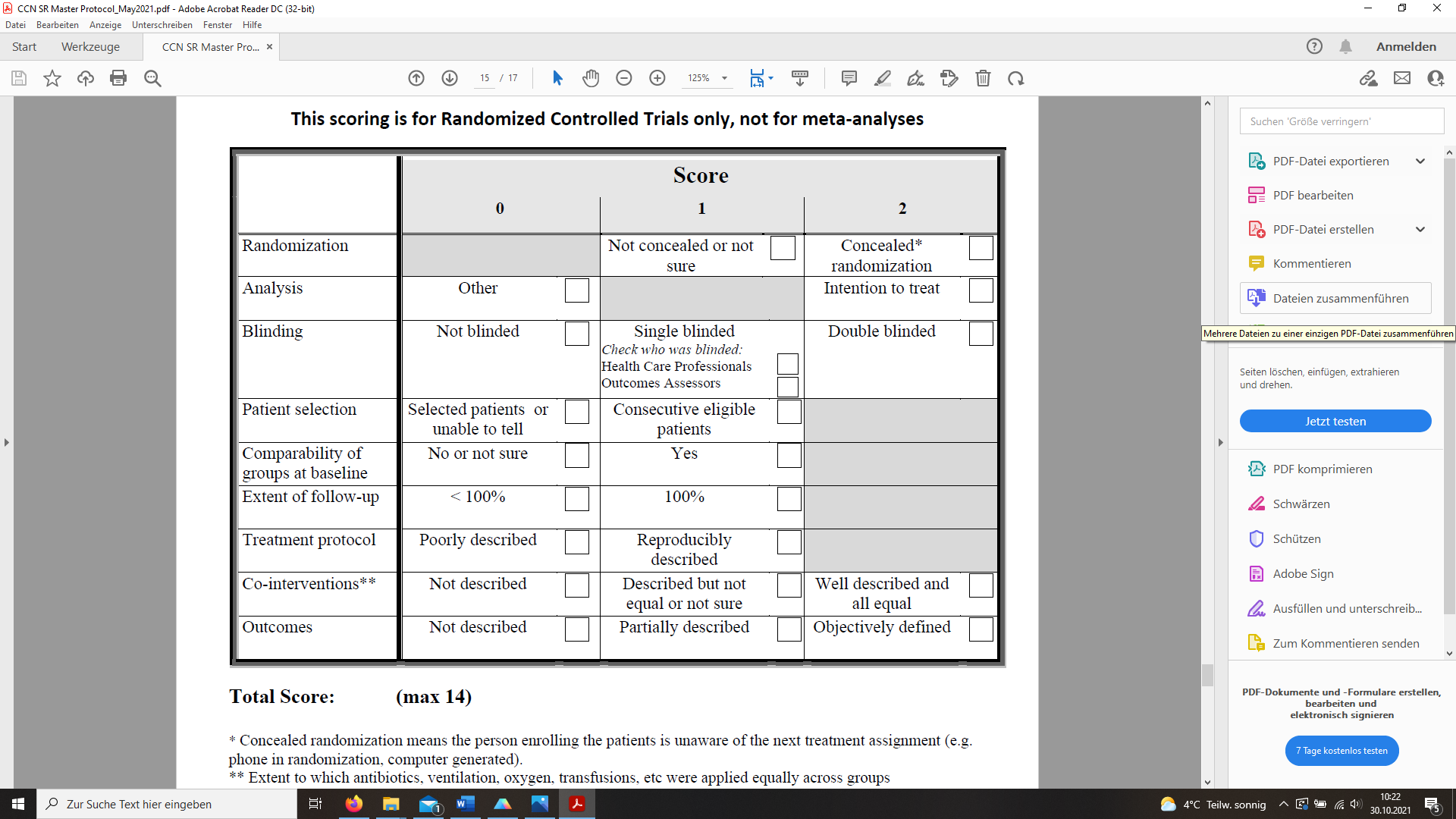

Supplement: Supplementary file 1 — Additional file 1. Methodological quality scoring system [file 13054_2022_3896_MOESM1_ESM.docx]
