# Supplementary material for: Omega-6 sparing effects of parenteral lipid emulsions—an updated systematic review and meta-analysis on clinical outcomes in critically ill patients
Source: Crit Care. 2022 Jan 19;26:23. doi: 10.1186/s13054-022-03896-3 (PMC8767697; doi:10.1186/s13054-022-03896-3)
Supplement: Supplementary file 2 — Additional file 2. List of excluded studies [file 13054_2022_3896_MOESM2_ESM.docx]

**Supplement 2. Excluded studies**(1-110)

Trials investigating non-ICU, elective surgery and cancer patients

1. Badía-Tahull MB, Llop-Talaverón JM, Leiva-Badosa E, et al: A randomised study on the clinical progress of high-risk elective major gastrointestinal surgery patients treated with olive oil-based parenteral nutrition with or without a fish oil supplement. *Br J Nutr* 2010; 104:737-741

2. Berger MM, Delodder F, Liaudet L, et al: Three short perioperative infusions of n-3 PUFAs reduce systemic inflammation induced by cardiopulmonary bypass surgery: a randomized controlled trial. *The American journal of clinical nutrition* 2013; 97:246-254

3. Berger MM, Tappy L, Revelly JP, et al: Fish oil after abdominal aorta aneurysm surgery. *Eur J Clin Nutr* 2008; 62:1116-1122

4. Cano NJ, Saingra Y, Dupuy AM, et al: Intradialytic parenteral nutrition: comparison of olive oil versus soybean oil-based lipid emulsions. *Br J Nutr* 2006; 95:152-159

5. Chambrier C, Guiraud M, Gibault JP, et al: Medium- and long-chain triacylglycerols in postoperative patients: structured lipids versus a physical mixture. *Nutrition* 1999; 15:274-277

6. Chen FM, Wang JY, Sun LC, et al: Efficacy of medium-chain triglycerides compared with long-chain triglycerides in total parenteral nutrition in patients with digestive tract cancer undergoing surgery. *Kaohsiung J Med Sci* 2005; 21:487-494

7. Demirer S, Sapmaz A, Karaca AS, et al: Effects of postoperative parenteral nutrition with different lipid emulsions in patients undergoing major abdominal surgery. *Ann Surg Treat Res* 2016; 91:309-315

8. Fiaccadori E, Tortorella G, Gonzi G, et al: Hemodynamic and respiratory effects of medium-chain and long-chain triglyceride fat emulsions: a prospective, randomized study. *Riv Ital Nutrz Parent Ent* 1997; 15:6-14

9. Furukawa K, Tashiro T, Yamamori H, et al: Effects of soybean oil emulsion and eicosapentaenoic acid on stress response and immune function after a severely stressful operation. *Ann Surg* 1999; 229:255-261

10. Gong Y, Liu Z, Liao Y, et al: Effectiveness of ω-3 Polyunsaturated Fatty Acids Based Lipid Emulsions for Treatment of Patients after Hepatectomy: A Prospective Clinical Trial. *Nutrients* 2016; 8

11. Grau T, Ruiz de Adana JC, Zubillaga S, et al: [Randomized study of two different fat emulsions in total parenteral nutrition of malnourished surgical patients;effect of infectious morbidity and mortality]. *Nutr Hosp* 2003; 18:159-166

12. Grimm H, Mertes N, Goeters C, et al: Improved fatty acid and leukotriene pattern with a novel lipid emulsion in surgical patients. *Eur J Nutr* 2006; 45:55-60

13. Hailer S, Jauch KW, Wolfram G: Influence of different fat emulsions with 10 or 20% MCT/LCT or LCT on lipoproteins in plasma of patients after abdominal surgery. *Ann Nutr Metab* 1998; 42:170-180

14. Hajdú N, Belágyi T, Issekutz A, et al: [Intravenous glutamine and early nasojejunal nutrition in severe acute pancreatitis -- a prospective randomized clinical study]. *Magy Seb* 2012; 65:44-51

15. Han YY, Lai SL, Ko WJ, et al: Effects of fish oil on inflammatory modulation in surgical intensive care unit patients. *Nutrition in clinical practice : official publication of the American Society for Parenteral and Enteral Nutrition* 2012; 27:91-98

16. Heidt MC, Vician M, Stracke SK, et al: Beneficial effects of intravenously administered N-3 fatty acids for the prevention of atrial fibrillation after coronary artery bypass surgery: a prospective randomized study. *Thorac Cardiovasc Surg* 2009; 57:276-280

17. Heller AR, Fischer S, Rössel T, et al: Impact of n-3 fatty acid supplemented parenteral nutrition on haemostasis patterns after major abdominal surgery. *Br J Nutr* 2002; 87 Suppl 1:S95-101

18. Heller AR, Rössel T, Gottschlich B, et al: Omega-3 fatty acids improve liver and pancreas function in postoperative cancer patients. *Int J Cancer* 2004; 111:611-616

19. Hutchinson M, Clemans G: Prospective trial of Liposyn® 20% in patients undergoing bone marrow transplantation. *Clinical Nutrition* 1984; 3:5-9

20. Jia Z-Y, Yang J, Xia Y, et al: Safety and efficacy of an olive oil-based triple-chamber bag for parenteral nutrition: a prospective, randomized, multi-center clinical trial in China. *Nutrition journal* 2015; 14:1-15

21. Jiang ZM, Wilmore DW, Wang XR, et al: Randomized clinical trial of intravenous soybean oil alone versus soybean oil plus fish oil emulsion after gastrointestinal cancer surgery. *Br J Surg* 2010; 97:804-809

22. Kłek S, Kulig J, Szczepanik AM, et al: The clinical value of parenteral immunonutrition in surgical patients. *Acta Chir Belg* 2005; 105:175-179

23. Kuse ER, Kotzerke J, Müller S, et al: Hepatic reticuloendothelial function during parenteral nutrition including an MCT/LCT or LCT emulsion after liver transplantation - a double-blind study. *Transpl Int* 2002; 15:272-277

24. Liang B, Wang S, Ye YJ, et al: Impact of postoperative omega-3 fatty acid-supplemented parenteral nutrition on clinical outcomes and immunomodulations in colorectal cancer patients. *World J Gastroenterol* 2008; 14:2434-2439

25. Linseisen J, Hoffmann J, Lienhard S, et al: Antioxidant status of surgical patients receiving TPN with an omega-3-fatty acid-containing lipid emulsion supplemented with alpha-tocopherol. *Clinical nutrition (Edinburgh, Scotland)* 2000; 19:177-184

26. Makay O, Kaya T, Firat O, et al: ω-3 Fatty acids have no impact on serum lactate levels after major gastric cancer surgery. *JPEN Journal of parenteral and enteral nutrition* 2011; 35:488-492

27. Martínez-Lozano Aranaga F, Gómez Ramos MJ, Sánchez Álvarez MdC: Eficacia y seguridad de dos emulsiones lipídicas de nutrición parenteral en pacientes críticos posquirúrgicos: Clinoleic® frente a SMOFlipid®. *Nutrición Hospitalaria* 2021; 38:5-10

28. Mertes N, Grimm H, Fürst P, et al: Safety and efficacy of a new parenteral lipid emulsion (SMOFlipid) in surgical patients: a randomized, double-blind, multicenter study. *Ann Nutr Metab* 2006; 50:253-259

29. Metry A, Abdelaal W, Ragaei M, et al: SMOFlipid versus intralipid in postoperative ICU patients. *Enliven: Journal of Anesthesiology and Critical Care Medicine* 2014; 1:15

30. Piper SN, Schade I, Beschmann RB, et al: Hepatocellular integrity after parenteral nutrition: comparison of a fish-oil-containing lipid emulsion with an olive-soybean oil-based lipid emulsion. *Eur J Anaesthesiol* 2009; 26:1076-1082

31. Puiggròs C, Sánchez J, Chacón P, et al: Evolution of lipid profile, liver function, and pattern of plasma fatty acids according to the type of lipid emulsion administered in parenteral nutrition in the early postoperative period after digestive surgery. *JPEN Journal of parenteral and enteral nutrition* 2009; 33:501-512

32. Salazar E, Alenezi S, Schwenger KJP, et al: Olive oil-based lipid emulsion is noninferior to soybean oil-based lipid emulsion in the acute care setting: A double-blind randomized controlled trial. *Nutrition* 2021; 89:111283

33. Senkal M, Geier B, Hannemann M, et al: Supplementation of omega-3 fatty acids in parenteral nutrition beneficially alters phospholipid fatty acid pattern. *JPEN Journal of parenteral and enteral nutrition* 2007; 31:12-17

34. Shan Y-S, Huang S-F, Wu Y-H, et al: Evaluation of the stability and safety of Venolipid MCT/LCT 20% administered by an all-in-one system in patients after major gastrointestinal surgery. *e-SPEN, the European e-Journal of Clinical Nutrition and Metabolism* 2008; 3:e135-e141

35. Wachtler P, König W, Senkal M, et al: Influence of a total parenteral nutrition enriched with omega-3 fatty acids on leukotriene synthesis of peripheral leukocytes and systemic cytokine levels in patients with major surgery. *The Journal of trauma* 1997; 42:191-198

36. Wang X, Li W, Li N, et al: Omega-3 fatty acids-supplemented parenteral nutrition decreases hyperinflammatory response and attenuates systemic disease sequelae in severe acute pancreatitis: a randomized and controlled study. *JPEN Journal of parenteral and enteral nutrition* 2008; 32:236-241

37. Weiss G, Meyer F, Matthies B, et al: Immunomodulation by perioperative administration of n-3 fatty acids. *Br J Nutr* 2002; 87 Suppl 1:S89-94

38. Wendel M, Rössel T, Bergmann S, et al: Impact of total parenteral nutrition including omega-3 fatty acids on the regulation of plasma lipoproteins and glycemic control after major abdominal surgery. *e-SPEN, the European e-Journal of Clinical Nutrition and Metabolism* 2007; 2:e103-e110

39. Wichmann MW, Thul P, Czarnetzki HD, et al: Evaluation of clinical safety and beneficial effects of a fish oil containing lipid emulsion (Lipoplus, MLF541): data from a prospective, randomized, multicenter trial. *Critical care medicine* 2007; 35:700-706

40. Zhu MW, Tang DN, Hou J, et al: Impact of fish oil enriched total parenteral nutrition on elderly patients after colorectal cancer surgery. *Chin Med J (Engl)* 2012; 125:178-181

Trials not reporting the clinical endpoints of our meta-analysis

41. Antébi H, Mansoor O, Ferrier C, et al: Liver function and plasma antioxidant status in intensive care unit patients requiring total parenteral nutrition: comparison of 2 fat emulsions. *JPEN Journal of parenteral and enteral nutrition* 2004; 28:142-148

42. Ball MJ: Parenteral nutrition in the critically ill: use of a medium chain triglyceride emulsion. *Intensive care medicine* 1993; 19:89-95

43. Calon B, Pottecher T, Frey A, et al: Long-chain versus medium and long-chain triglyceride-based fat emulsion in parental nutrition of severe head trauma patients. *Infusionstherapie* 1990; 17:246-248

44. Chacón Castro MP, Jiménez Sesé G, Salvadó Salvát J, et al: [The effect of fatty emulsions with distinct triglyceride compositions on the lipid metabolism of the septic patient]. *Nutr Hosp* 2000; 15:13-17

45. Chassard D, Guiraud M, Gauthier J, et al: Effects of intravenous medium-chain triglycerides on pulmonary gas exchanges in mechanically ventilated patients. *Critical care medicine* 1994; 22:248-251

46. Chen H-z, Cai L, Zhang X: Comparison of the effects of structured triglyceride and medium/long chain triglycerides on liver function and serum lipid in critical patients. *China Pharmacy* 2013; 24:2238-2240

47. Diboune M, Ferard G, Ingenbleek Y, et al: Composition of phospholipid fatty acids in red blood cell membranes of patients in intensive care units: effects of different intakes of soybean oil, medium-chain triglycerides, and black-currant seed oil. *JPEN Journal of parenteral and enteral nutrition* 1992; 16:136-141

48. García-de-Lorenzo A, López-Martínez J, Planas M, et al: Safety and metabolic tolerance of a concentrated long-chain triglyceride lipid emulsion in critically ill septic and trauma patients. *JPEN Journal of parenteral and enteral nutrition* 2003; 27:208-215

49. Hwang TL, Huang SL, Chen MF: Effects of intravenous fat emulsion on respiratory failure. *Chest* 1990; 97:934-938

50. Jarnberg P: Liposyn® versus Intralipid®. A comparative study of two lipid emulsions in critically ill patients receiving total parenteral nutrition. *Current Therapeutic Research-Clinical and Experimental* 1991; 50:38-44

51. Jeevanandam M, Holaday NJ, Voss T, et al: Efficacy of a mixture of medium-chain triglyceride (75%) and long-chain triglyceride (25%) fat emulsions in the nutritional management of multiple-trauma patients. *Nutrition* 1995; 11:275-284

52. Kalfarentzos F, Kokkinis K, Leukaditi K, et al: Comparison between two fat emulsions: Intralipid 30 cent vs intralipid 10 cent in critically ill patients. *Clinical nutrition (Edinburgh, Scotland)* 1998; 17:31-34

53. Köller M, Senkal M, Kemen M, et al: Impact of omega-3 fatty acid enriched TPN on leukotriene synthesis by leukocytes after major surgery. *Clinical nutrition (Edinburgh, Scotland)* 2003; 22:59-64

54. Maeshima Y, Fukatsu K, Moriya T, et al: Influence of adding fish oil to parenteral nutrition on gut-associated lymphoid tissue. *JPEN Journal of parenteral and enteral nutrition* 2007; 31:416-422

55. Manuel-y-Keenoy B, Nonneman L, De Bosscher H, et al: Effects of intravenous supplementation with alpha-tocopherol in patients receiving total parenteral nutrition containing medium- and long-chain triglycerides. *Eur J Clin Nutr* 2002; 56:121-128

56. Masclans JR, Iglesia R, Bermejo B, et al: Gas exchange and pulmonary haemodynamic responses to fat emulsions in acute respiratory distress syndrome. *Intensive care medicine* 1998; 24:918-923

57. Mayer K, Fegbeutel C, Hattar K, et al: Omega-3 vs. omega-6 lipid emulsions exert differential influence on neutrophils in septic shock patients: impact on plasma fatty acids and lipid mediator generation. *Intensive care medicine* 2003; 29:1472-1481

58. Mayer K, Gokorsch S, Fegbeutel C, et al: Parenteral nutrition with fish oil modulates cytokine response in patients with sepsis. *American journal of respiratory and critical care medicine* 2003; 167:1321-1328

59. Mayer K, Meyer S, Reinholz-Muhly M, et al: Short-time infusion of fish oil-based lipid emulsions, approved for parenteral nutrition, reduces monocyte proinflammatory cytokine generation and adhesive interaction with endothelium in humans. *Journal of immunology (Baltimore, Md : 1950)* 2003; 171:4837-4843

60. Min L, Hongwei Y, Sun Y: Clinical application of the structured triglyceride and physical mixed MCT/LCT in critically ill patients. *Modern Medicine Journal of China* 2012; 14:8-10

61. Niu G, Zhao R, Gao F, et al: Effect of omega‐3 polyunsaturated fatty acids on intestinal mucosal barrier of patients with severe acute pancreatitis. *Zhongguo Linchuang Yingyang Zazhi [Chinese Journal of Clinical Nutrition]* 2014; 22:329-333

62. Pastó L, Pellicer E, Llopart R, et al: [Comparative study of two lipid emulsions in patients receiving total parenteral nutrition]. *Nutr Hosp* 1991; 6:29-33

63. Planas M, Masclans JR, Iglesia R, et al: Eicosanoids and fat emulsions in acute respiratory distress syndrome patients. *Nutrition* 1997; 13:202-205

64. Planas M, Porta I, Sagristá ML, et al: Fatty acid composition of platelet membrane lipids after administration of two different fat emulsions in critically ill patients. *Intensive care medicine* 1999; 25:395-398

65. Schauder P, Röhn U, Schäfer G, et al: Impact of fish oil enriched total parenteral nutrition on DNA synthesis, cytokine release and receptor expression by lymphocytes in the postoperative period. *Br J Nutr* 2002; 87 Suppl 1:S103-110

66. Smirniotis V, Kostopanagiotou G, Vassiliou J, et al: Long chain versus medium chain lipids in patients with ARDS: effects on pulmonary haemodynamics and gas exchange. *Intensive care medicine* 1998; 24:1029-1033

67. Su M, He L, Liu Z, et al: Applied studies of structured triglycerides for parenteral nutrition in severe hemorrhagic shock patients after resuscitation. *Zhonghua yi xue za zhi* 2012; 92:827-830

68. Sungurtekin H, Değirmenci S, Sungurtekin U, et al: Comparison of the effects of different intravenous fat emulsions in patients with systemic inflammatory response syndrome and sepsis. *Nutrition in clinical practice : official publication of the American Society for Parenteral and Enteral Nutrition* 2011; 26:665-671

69. Tappy L, Berger MM, Schwarz JM, et al: Metabolic effects of parenteral nutrition enriched with n-3 polyunsaturated fatty acids in critically ill patients. *Clinical nutrition (Edinburgh, Scotland)* 2006; 25:588-595

70. Widhalm K, Kohl S, Hammerle A: The clinical application of two newly developed lipid emulsions (Solipid 20% S&E) in critically ill patients. *Infusionsther Transfusionsmed* 1996; 23:8-12

71. Xiong J, Zhu S, Zhou Y, et al: Regulation of omega-3 fish oil emulsion on the SIRS during the initial stage of severe acute pancreatitis. *J Huazhong Univ Sci Technolog Med Sci* 2009; 29:35-38

Trials without randomized control, including systematic reviews, meta- or sub-analyses

72. Barros KV, Cassulino AP, Schalch L, et al: Pharmaconutrition: acute fatty acid modulation of circulating cytokines in elderly patients in the ICU. *JPEN Journal of parenteral and enteral nutrition* 2014; 38:467-474

73. Barros KV, Cassulino AP, Schalch L, et al: Supplemental intravenous n-3 fatty acids and n-3 fatty acid status and outcome in critically ill elderly patients in the ICU receiving enteral nutrition. *Clinical nutrition (Edinburgh, Scotland)* 2013; 32:599-605

74. Chen H, Wang S, Zhao Y, et al: Correlation analysis of omega-3 fatty acids and mortality of sepsis and sepsis-induced ARDS in adults: data from previous randomized controlled trials. *Nutr J* 2018; 17:57

75. Chen H, Wang W, Zhang H, et al: Treating effects of Omega-3 fish oil fatty acids on sepsis-induced MODS. *Guangdong Med J* 2011; 32:3271-3273

76. Chen J, Yan J, Cai GL, et al: Structured lipid emulsion as nutritional therapy for the elderly patients with severe sepsis. *Chin Med J (Engl)* 2013; 126:2329-2332

77. Chen W, Jiang H, Zhou ZY, et al: Is omega-3 fatty acids enriched nutrition support safe for critical ill patients? A systematic review and meta-analysis. *Nutrients* 2014; 6:2148-2164

78. Gazzaniga AB, Day AT, Sankary H: The efficacy of a 20 per cent fat emulsion as a peripherally administered substrate. *Surg Gynecol Obstet* 1985; 160:387-392

79. Hall TC, Bilku DK, Neal CP, et al: The impact of an omega-3 fatty acid rich lipid emulsion on fatty acid profiles in critically ill septic patients. *Prostaglandins Leukot Essent Fatty Acids* 2016; 112:1-11

80. Heller AR, Rössler S, Litz RJ, et al: Omega-3 fatty acids improve the diagnosis-related clinical outcome. *Critical care medicine* 2006; 34:972-979

81. Lu C, Sharma S, McIntyre L, et al: Omega-3 supplementation in patients with sepsis: a systematic review and meta-analysis of randomized trials. *Ann Intensive Care* 2017; 7:58

82. Lukach VN, Girsh AO, Tolkach AB, et al: [Effective use of third generation fat emulsion in total parenteral nutrition program in patients with severe sepsis]. *Anesteziol Reanimatol* 2011:30-34

83. Manzanares W, Dhaliwal R, Jurewitsch B, et al: Alternative lipid emulsions in the critically ill: a systematic review of the evidence. *Intensive care medicine* 2013; 39:1683-1694

84. Manzanares W, Dhaliwal R, Jurewitsch B, et al: Parenteral fish oil lipid emulsions in the critically ill: a systematic review and meta-analysis. *JPEN Journal of parenteral and enteral nutrition* 2014; 38:20-28

85. Manzanares W, Langlois PL, Dhaliwal R, et al: Intravenous fish oil lipid emulsions in critically ill patients: an updated systematic review and meta-analysis. *Critical care (London, England)* 2015; 19:167

86. Ni C, Cao J, Li D, et al: Parenteral nutrition effects of Omega-3 fatty acids on C-reactive protein, high-density lipoprotein, lymphocyte characteristics and the treatment of critically ill patients. *Cell Mol Biol (Noisy-le-grand)* 2020; 66:52-56

87. Palmer AJ, Ho CK, Ajibola O, et al: The role of ω-3 fatty acid supplemented parenteral nutrition in critical illness in adults: a systematic review and meta-analysis. *Critical care medicine* 2013; 41:307-316

88. Pradelli L, Klek S, Mayer K, et al: Omega-3 fatty acid-containing parenteral nutrition in ICU patients: systematic review with meta-analysis and cost-effectiveness analysis. *Critical care (London, England)* 2020; 24:634

89. Pradelli L, Mayer K, Klek S, et al: ω-3 Fatty-Acid Enriched Parenteral Nutrition in Hospitalized Patients: Systematic Review With Meta-Analysis and Trial Sequential Analysis. *JPEN Journal of parenteral and enteral nutrition* 2020; 44:44-57

90. Tao W, Li PS, Shen Z, et al: Effects of omega-3 fatty acid nutrition on mortality in septic patients: a meta-analysis of randomized controlled trials. *BMC Anesthesiol* 2016; 16:39

91. van der Meij BS, van Bokhorst-de van der Schueren MA, Langius JA, et al: n-3 PUFAs in cancer, surgery, and critical care: a systematic review on clinical effects, incorporation, and washout of oral or enteral compared with parenteral supplementation. *The American journal of clinical nutrition* 2011; 94:1248-1265

92. Wan X, Gao X, Bi J, et al: Use of n-3 PUFAs can decrease the mortality in patients with systemic inflammatory response syndrome: a systematic review and meta-analysis. *Lipids Health Dis* 2015; 14:23

93. Wu GH, Zaniolo O, Schuster H, et al: Structured triglycerides versus physical mixtures of medium- and long-chain triglycerides for parenteral nutrition in surgical or critically ill adult patients: Systematic review and meta-analysis. *Clinical nutrition (Edinburgh, Scotland)* 2017; 36:150-161

94. Zhu D, Zhang Y, Li S, et al: Enteral omega-3 fatty acid supplementation in adult patients with acute respiratory distress syndrome: a systematic review of randomized controlled trials with meta-analysis and trial sequential analysis. *Intensive care medicine* 2014; 40:504-512

Trials with treatment paradigms beyond the scope of our meta-analysis

95. Adams S, Yeh YY, Jensen GL: Changes in plasma and erythrocyte fatty acids in patients fed enteral formulas containing different fats. *JPEN Journal of parenteral and enteral nutrition* 1993; 17:30-34

96. Adolph M, Hailer S, Echart J: Serum phospholipid fatty acids in severely injured patients on total parenteral nutrition with medium chain/long chain triglyceride emulsions. *Ann Nutr Metab* 1995; 39:251-260

97. Battistella FD, Widergren JT, Anderson JT, et al: A prospective, randomized trial of intravenous fat emulsion administration in trauma victims requiring total parenteral nutrition. *The Journal of trauma* 1997; 43:52-58; discussion 58-60

98. Bernier J, Jobin N, Emptoz-Bonneton A, et al: Decreased corticosteroid-binding globulin in burn patients: relationship with interleukin-6 and fat in nutritional support. *Critical care medicine* 1998; 26:452-460

99. Gogos CA, Ginopoulos P, Salsa B, et al: Dietary omega-3 polyunsaturated fatty acids plus vitamin E restore immunodeficiency and prolong survival for severely ill patients with generalized malignancy: a randomized control trial. *Cancer* 1998; 82:395-402

100. Hosny M, Nahas R, Ali S, et al: Impact of oral omega-3 fatty acids supplementation in early sepsis on clinical outcome and immunomodulation. *The Egyptian Journal of Critical Care Medicine* 2013; 1:119-126

101. Huschak G, Zur Nieden K, Hoell T, et al: Olive oil based nutrition in multiple trauma patients: a pilot study. *Intensive care medicine* 2005; 31:1202-1208

102. Kari A, Hersio K, Takala J, et al: Comparison of two long-chain triglyceride fat emulsions in parenteral nutrition of critically ill patients. *Current therapeutic research* 1989; 45:1077-1087

103. Lekka ME, Liokatis S, Nathanail C, et al: The impact of intravenous fat emulsion administration in acute lung injury. *American journal of respiratory and critical care medicine* 2004; 169:638-644

104. Najmi M, Vahdat Shariatpanahi Z, Tolouei M, et al: Effect of oral olive oil on healing of 10-20% total body surface area burn wounds in hospitalized patients. *Burns* 2015; 41:493-496

105. Sabater J, Masclans JR, Sacanell J, et al: Effects on hemodynamics and gas exchange of omega-3 fatty acid-enriched lipid emulsion in acute respiratory distress syndrome (ARDS): a prospective, randomized, double-blind, parallel group study. *Lipids Health Dis* 2008; 7:39

106. Suchner U, Katz DP, Fürst P, et al: Effects of intravenous fat emulsions on lung function in patients with acute respiratory distress syndrome or sepsis. *Critical care medicine* 2001; 29:1569-1574

107. Taftachi F, Sanaei-Zadeh H, Sepehrian B, et al: Lipid emulsion improves Glasgow coma scale and decreases blood glucose level in the setting of acute non-local anesthetic drug poisoning--a randomized controlled trial. *Eur Rev Med Pharmacol Sci* 2012; 16 Suppl 1:38-42

108. Theilla M, Singer P, Cohen J, et al: A diet enriched in eicosapentanoic acid, gamma-linolenic acid and antioxidants in the prevention of new pressure ulcer formation in critically ill patients with acute lung injury: A randomized, prospective, controlled study. *Clinical nutrition (Edinburgh, Scotland)* 2007; 26:752-757

109. Yan H, Huang X, Xiao K, et al: Effects of medium and long-chain triglyceride on the immune function of burn patients during early postburn stage. *Zhonghua shao shang za zhi= Zhonghua shaoshang zazhi= Chinese journal of burns* 2003; 19:202-205

No fulltext available

110. Liang X, Fang L, Zhang Y: Inhibitive effects of Omega-3 fish oil to severe sepsis. *China Prac Med* 2009; 4:158-159
