# Supplementary material for: Omega-6 sparing effects of parenteral lipid emulsions—an updated systematic review and meta-analysis on clinical outcomes in critically ill patients
Source: Crit Care. 2022 Jan 19;26:23. doi: 10.1186/s13054-022-03896-3 (PMC8767697; doi:10.1186/s13054-022-03896-3)
Supplement: Supplementary file 3 — Additional file 3. List of included studies, outcomes and bias risk assessment [file 13054_2022_3896_MOESM3_ESM.docx]

Supplement 3. Included studies, bias risk assessment and outcomes

# Study Intervention Included outcomes

**ICU population**

| **PN trials: omega-6 reduced formulations vs. standard care lipid emulsions** | | | | | | | | |
| --- | --- | --- | --- | --- | --- | --- | --- | --- |
| Nijveldt *et al.*, 1998 | 20 |  |  |  | 10 (II) | Sepsis, Trauma | SO/MCT vs. SO | Overall mortality (not specified), Duration of MV |
| Lindgren *et al.*, 2001 | 20 |  |  |  | 8 (II) | Sepsis, Trauma | SO/MCT vs. SO | Overall mortality (hospital mortality), ICU LOS |
| Garnacho-Montero *et al.*, 2002 | 72 |  |  |  | 4 (II) | Sepsis | SO/MCT vs. SO | Overall mortality (hospital mortality), ICU LOS |
| Grecu *et al.*, 2003^a^ | 54 |  |  |  | 12 (I) | Abdominal sepsis | SO/MCT/FO (Omegaven) vs. SO/MCT | Overall mortality (ICU mortality), ICU LOS, Hospital LOS, Duration of MV, Infections (pneumonia) |
| García-de-Lorenzo *et al.*, 2005 | 22 |  |  |  | 10 (II) | Burn injuries | SO/OO vs. SO/MCT | Overall mortality (hospital mortality), ICU LOS, Hospital LOS, Duration of MV, Infections (pneumonia, bacteremia, infection of the burn area) |
| Iovinelli *et al.*, 2007 | 14 |  |  |  | 4 (II) | COPD | SO/MCT vs. SO | Overall mortality (15 Days), Duration of MV |
| Friesecke *et al.*, 2008 | 165 |  |  |  | 10 (I) | Medical ICU | SO/MCT/FO (Omegaven) vs. SO/MCT | Overall mortality (28-day mortality), 28-day mortality, ICU LOS, duration of MV, infections (pneumonia, urinary tract infection, catheter-related bloodstream infection) |
| Guo *et al.*, 2008 | 80 |  |  |  | 4 (II) | Sepsis | SO/MCT/FO (other) vs. SO/MCT | Overall mortality (28-day mortality), 28-day mortality, ICU LOS |
| Wang *et al.*, 2009 | 56 |  |  |  | 11 (II) | Pancreatitis | SO/MCT/FO (Omegaven) vs. SO/MCT | Overall mortality (ICU mortality), Infections (pneumonia, sepsis, abscess) |
| Qu *et al.*, 2009 | 40 |  |  |  | 5 (II) | Sepsis | Routine PN (no details)/FO (other) vs. Routine PN | Overall mortality (28-day mortality), 28-day mortality |
| Barbosa *et al.*, 2010 | 23 |  |  |  | 10 (II) | SIRS, Sepsis | SO/MCT/FO (other) vs. SO/MCT | Overall mortality (28-day mortality), 28-day mortality, ICU LOS, Hospital LOS, Duration of MV |
| Sabater *et al.*, 2011 |  |  |  |  | 9 (II) | ARDS | SO/MCT/FO (other) vs. SO | Overall mortality (not specified) |
| Pontes-Arruda *et al.*, 2012^a, b^ | 204 |  |  |  | 9 (II) | Mostly surgical ICU | SO/OO vs. SO/MCT | Overall mortality (28-day mortality), 28-day mortality, Infections (pneumonia, bloodstream infection,  catheter-related bloodstream infection, unclassified ICU-acquired infection)^c^ |
| Umpierrez *et al.*, 2012 | 100 |  |  |  | 14 (I) | Mostly surgical ICU | SO/OO vs. SO | Overall mortality (hospital mortality), ICU LOS, Hospital LOS, Infections (pneumonia, urinary tract infection, bacteremia, wound infection) |
| Gultekin *et al.*, 2014 | 32 |  |  |  | 7 (II) | Sepsis | SO/OO/FO (Omegaven) vs. SO/OO | Overall mortality (hospital mortality), Hospital LOS |
| Wang *et al.*, 2014 | 53 |  |  |  | 6 (II) | Abdominal sepsis | Routine PN (no details)/FO (Omegaven) vs. Routine PN | ICU LOS, Duration of MV |
| Grau-Carmona *et al.*, 2015^a^ | 159 |  |  |  | 10 (I) | Medical and surgical ICU | SO/MCT/FO (other) vs. SO/MCT | Overall mortality (hospital mortality), ICU LOS, Hospital LOS, Duration of MV, Infections (pneumonia, urinary tract infection, bacteremia, wound infection, abscess) |
| Chen *et al.*, 2017a | 78 |  |  |  | 9 (II) | Sepsis | SO/FO (Omegaven) vs. SO | Overall mortality (28-day mortality), 28-day mortality |
| Chen *et al.*, 2017b | 48 |  |  |  | 9 (II) | SIRS | Routine PN (no details)/FO (Omegaven) vs. Routine PN | Overall mortality (28-day mortality), 28-day mortality, ICU LOS |
| Donoghue *et al.*, 2019^a^ | 68 |  |  |  | 7 (II) | SIRS, Sepsis, ARDS | SO/MCT/OO/FO (other) vs. SO | Overall mortality (ICU mortality), ICU LOS, Duration of MV |
| Singer *et al.*, 2021 | 95 |  |  |  | 9 (II) | MV | Supplemental PN: SO/MCT/FO (other)  vs. SO/MCT^d^ | Overall mortality (28-day mortality), 28-day mortality^c^ |

| **Stand-alone FO supplement vs. standard care** | | | | | | | | |
| --- | --- | --- | --- | --- | --- | --- | --- | --- |
| Gupta *et al.*, 2011 | 61 |  |  |  | 9 (I) | Suspected ARDS | Standard care/FO (Omegaven) vs. Standard care | Overall mortality (28-day mortality), 28-day mortality, ICU LOS, Hospital LOS, Duration of MV |
| Khor *et al.*, 2011 | 27 |  |  |  | 8 (II) | Sepsis | Standard care/FO (Omegaven)  vs. Standard care/Placebo (normal saline) | ICU LOS, Hospital LOS |
| Zhao *et al.*, 2011 | 116 |  |  |  | 5 (II) | Sepsis | Standard care/FO (Omegaven) vs. Standard care | Overall mortality (28-day mortality), 28-day mortality, ICU LOS |
| Burkhart *et al.*, 2014 | 50 |  |  |  | 9 (II) | Sepsis | Standard care/FO (Omegaven) vs. Standard care | Overall mortality (109 days)^c^ |
| Hall *et al.*, 2015 | 60 |  |  |  | 10 (II) | Sepsis | Standard care/FO (Omegaven) vs. Standard care | Overall mortality (28-day mortality), 28-day mortality, ICU LOS, Hospital LOS, Infections (secondary infection, not further classified) |

ARDS, acute respiratory distress syndrome; COPD, chronic obstructive pulmonary disease; FO, fish oil; ICU, intensive care unit; LOS, length of stay; MCT, medium chain triglycerides; MV, mechanical ventilation; OO, olive oil; PN, parenteral nutrition; SIRS, systemic inflammatory response syndrome; SO, soybean oil. Assessment for methodological quality and bias risk (Canadian Nutrition Support Clinical Practice Guidelines): green = criteria fullfilled; yellow = not sure about the randomization or single blinded instead of double blinded; red = criteria not fullfilled.

^a^ Additional information were retrieved from the authors. The study by Grecu *et al.* was only published as an abstract.

^b^ This study included an additional treatment group to evaluate the effects of multichambered bags in PN, which was excluded for our meta-analysis.

^c^ Further outcomes were reported, however continuous data were not available as mean and standard deviation.

^d^ Patients additionally received enteral nutrition, including enteral FO in the experimental group. Sensitivity analyses revealed that the overall results were not significantly altered after excluding this trial.
