# Supplementary material for: Omega-6 sparing effects of parenteral lipid emulsions—an updated systematic review and meta-analysis on clinical outcomes in critically ill patients
Source: Crit Care. 2022 Jan 19;26:23. doi: 10.1186/s13054-022-03896-3 (PMC8767697; doi:10.1186/s13054-022-03896-3)
Supplement: Supplementary file 5 — Additional file 5. Length of mechanical ventilation in trials using an omega-6 fatty acid reducing strategy [file 13054_2022_3896_MOESM5_ESM.docx]

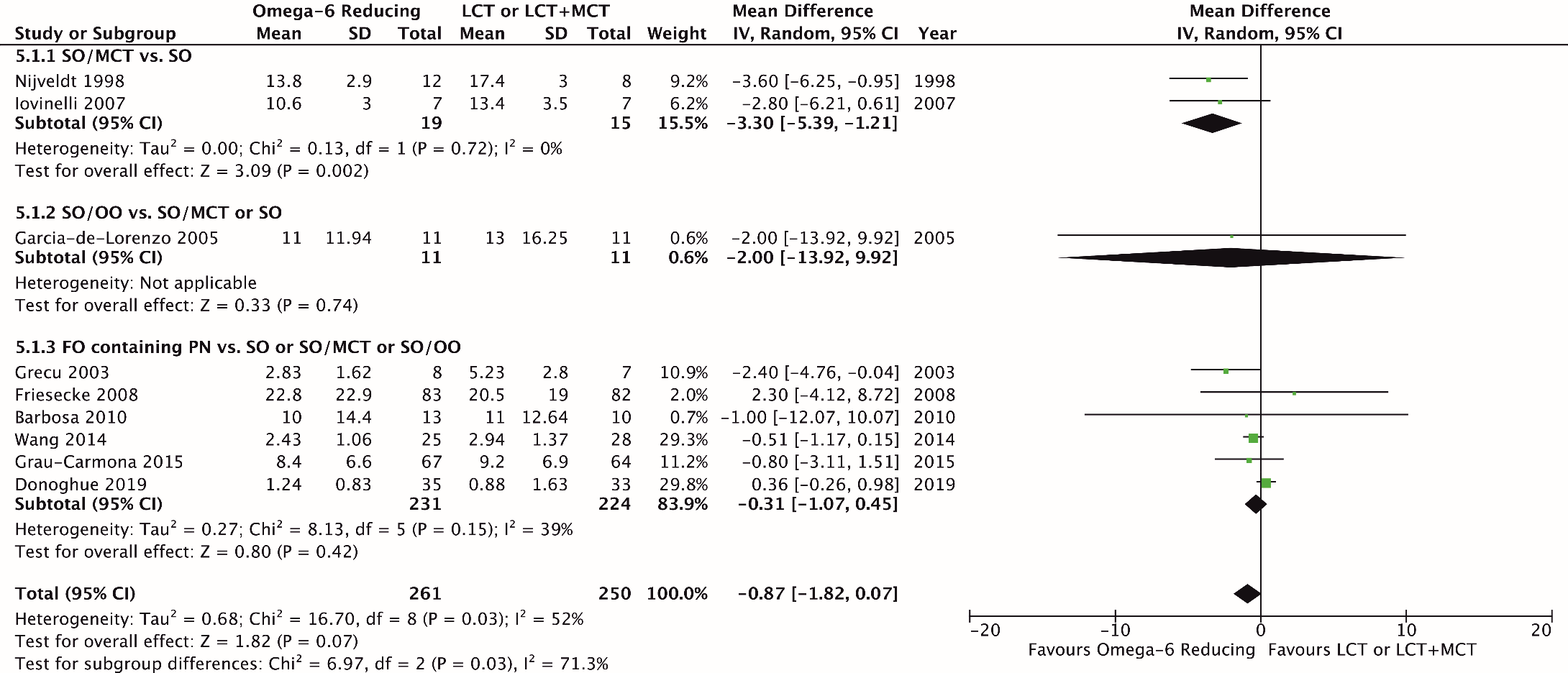


**Supplement 5:** Length of mechanical ventilation in trials using an omega-6 fatty acid reducing strategy.
